# Studying the post-COVID-19 condition: research challenges, strategies, and importance of Core Outcome Set development

**DOI:** 10.1186/s12916-021-02222-y

**Published:** 2022-02-04

**Authors:** Daniel Munblit, Timothy R. Nicholson, Dale M. Needham, Nina Seylanova, Callum Parr, Jessica Chen, Alisa Kokorina, Louise Sigfrid, Danilo Buonsenso, Shinjini Bhatnagar, Ramachandran Thiruvengadam, Ann M. Parker, Jacobus Preller, Sergey Avdeev, Frederikus A. Klok, Allison Tong, Janet V. Diaz, Wouter De Groote, Nicoline Schiess, Athena Akrami, Frances Simpson, Piero Olliaro, Christian Apfelbacher, Regis Goulart Rosa, Jennifer R. Chevinsky, Sharon Saydah, Jochen Schmitt, Alla Guekht, Sarah L. Gorst, Jon Genuneit, Luis Felipe Reyes, Alan Asmanov, Margaret E. O’Hara, Janet T. Scott, Melina Michelen, Charitini Stavropoulou, John O. Warner, Margaret Herridge, Paula R. Williamson

**Affiliations:** 1 Department of Paediatrics and Paediatric Infectious Diseases, Institute of Child’s Health, Sechenov First Moscow State Medical University (Sechenov University), Moscow, Russia; 2 Inflammation, Repair and Development Section, National Heart and Lung Institute, Faculty of Medicine, Imperial College London, London, UK; 3 Research and Clinical Center for Neuropsychiatry, Moscow, Russia; 4 Institute of Psychiatry, Psychology and Neuroscience, King’s College London, London, UK; 5 Outcomes After Critical Illness and Surgery (OACIS) Research Group, Johns Hopkins University, Baltimore, MD USA; 6 Pulmonary and Critical Care Medicine, Department of Medicine, Johns Hopkins University School of Medicine, Baltimore, MD USA; 7 Physical Medicine and Rehabilitation, Johns Hopkins University School of Medicine, Baltimore, MD USA; 8 Sechenov Biomedical Science and Technology Park, Sechenov First Moscow State Medical University (Sechenov University), Moscow, Russia; 9 Faculty of Medicine, Imperial College London, London, UK; 10 Pirogov Russian National Research Medical University, Moscow, Russia; 11 ISARIC Global Support Centre, Centre for Tropical Medicine and Global Health, University of Oxford, Oxford, UK; 12 Department of Woman and Child Health and Public Health, Fondazione Policlinico Universitario A. Gemelli IRCCS, Rome, Italy; 13 Dipartimento di Scienze Biotecnologiche di Base, Cliniche Intensivologiche e Perioperatorie, Università Cattolica del Sacro Cuore, Rome, Italy; 14 Global Health Research Institute, Istituto di Igiene, Università Cattolica del Sacro Cuore, Roma, Italy; 15 Maternal and Child Health Program, Translational Health Science and Technology Institute, Faridabad, Delhi, National Capital Region India; 16 Clinical Management, WHO, WHE, Geneva, Switzerland; 17 Department of Pulmonology, Sechenov First Moscow State Medical University (Sechenov University), Moscow, Russia; 18 Department of Medicine - Thrombosis and Hemostasis, Leiden University Medical Center, Leiden, the Netherlands; 19 Sydney School of Public Health, The University of Sydney, Sydney, Australia; 20 NCD Department, Rehabilitation Programme, WHO, Geneva, Switzerland; 21 WHO Brain Health Unit, Geneva, Switzerland; 22 Sainsbury Wellcome Centre, UCL, London, UK; 23 Patient-Led Research Collaborative, Washington, DC USA; 24 Coventry University, Coventry, UK; 25 ISARIC Global Support Centre, Nuffield Department of Medicine, University of Oxford, Oxford, UK; 26 Institute of Social Medicine and Health Systems Research, Faculty of Medicine, Otto von Guericke University Magdeburg, Magdeburg, Germany; 27 Critical Care Department, Hospital Moinhos de Vento, Porto Alegre, Brazil; 28 Brazilian Research in Intensive Care Network (BRICNet), São Paulo, Brazil; 29 COVID-19 Response Team, Centers for Disease Control and Prevention, Atlanta, GA USA; 30 Epidemic Intelligence Service, Centers for Disease Control and Prevention, Atlanta, GA USA; 31 Respiratory Viruses Branch, Division of Viral Diseases, Centers for Disease Control and Prevention, Atlanta, GA USA; 32 Center for Evidence-Based Healthcare, Medical Faculty Carl Gustav Carus, TU Dresden, Dresden, Germany; 33 Department of Health Data Science, University of Liverpool, Liverpool, UK; 34 Paediatric Epidemiology, Department of Pediatrics, Medical Faculty, Leipzig University, Leipzig, Germany; 35 Universidad de La Sabana, Chía, Colombia; 36 Clínica Universidad de La Sabana, Chía, Colombia; 37 The Research and Clinical Institute for Pediatrics named after Academician Yuri Veltischev of the Pirogov Russian National Research Medical University, Moscow, Russia; 38 Long COVID Support, London, UK; 39 MRC-University of Glasgow, Centre for Virus Research, Glasgow, UK; 40 School of Health Sciences, City, University of London, London, UK; 41 Paediatric Infectious Diseases, Imperial College Healthcare NHS Trust, London, UK; 42 Interdepartmental Division of Critical Care Medicine, University of Toronto, Toronto, ON Canada; 43 Department of Medicine, University Health Network, Toronto, ON Canada; 44 MRC/NIHR Trials Methodology Research Partnership, Department of Health Data Science, University of Liverpool (a member of Liverpool Health Partners), Liverpool, UK

**Keywords:** COVID-19, COVID-19 sequalae, Long COVID, Post-acute sequelae of SARS-CoV-2 infection, PASC, Post-COVID-19 condition, Outcomes, Core Outcome Set

## Abstract

**Background:**

A substantial portion of people with COVID-19 subsequently experience lasting symptoms including fatigue, shortness of breath, and neurological complaints such as cognitive dysfunction many months after acute infection. Emerging evidence suggests that this condition, commonly referred to as *long COVID* but also known as *post-acute sequelae of SARS-CoV-2 infection (PASC)* or *post-COVID-19 condition*, could become a significant global health burden.

**Main text:**

While the number of studies investigating the *post-COVID-19 condition* is increasing, there is no agreement on how this new disease should be defined and diagnosed in clinical practice and what relevant outcomes to measure. There is an urgent need to optimise and standardise outcome measures for this important patient group both for clinical services and for research and to allow comparing and pooling of data.

**Conclusions:**

A Core Outcome Set for *post-COVID-19 condition* should be developed in the shortest time frame possible, for improvement in data quality, harmonisation, and comparability between different geographical locations. We call for a global initiative, involving all relevant partners, including, but not limited to, healthcare professionals, researchers, methodologists, patients, and caregivers. We urge coordinated actions aiming to develop a Core Outcome Set (COS) for *post-COVID-19 condition* in both the adult and paediatric populations.

## Background

The coronavirus disease 2019 (COVID-19) pandemic has necessitated rapid responses from healthcare systems and research networks globally. Although a large amount of comprehensive data on acute symptoms and clinical management has been collected and analysed, there are currently no established clinical definition or Core Outcome Sets (COS). Indeed, even the terminology of the condition is debated with variable terms and definitions for the post-COVID-19 condition including *long COVID*, “long haulers”, *post-acute sequelae of SARS-CoV-2 infection (PASC)*, or *post-COVID-19 condition*, the term used by the World Health Organization (WHO). With over 217 million confirmed COVID-19 cases globally [1], *post-COVID-19 condition* risks affecting millions of people worldwide, making it an urgent research priority [2]. Although wide-scale vaccination may eventually lead to a decline in the number of COVID-19 cases, with cases rising worldwide, the pandemic is far from over. There is an urgent need for consensus on critically important core outcomes to be measured in *post-COVID-19 condition*. Establishing a COS will ensure that critically important outcomes are measured and reported in a consistent manner in research and practice settings. The consistent use of the most important outcomes across studies and clinical practice is essential to compare and collate the research findings with translation into clinical recommendations for patient care.

In this manuscript, we discuss the existing data known about *post-COVID-19 condition* research following WHO’s systematic approach to identifying research gaps [3] with the principal purpose of suggesting and outlining the implementation of a COS for the *post-COVID-19 condition* (i) to allow for the assessment of outcomes which are of the greatest relevance and importance to stakeholders and relevant target populations including patients, families, clinicians, researchers, health systems, public health policymakers, industry, and funding organisations; (ii) to improve the consistency and quality of data collection; and (iii) to build a foundation for data sharing for pooled analysis for meta-analysis and comparison of results across studies and global regions.

Given the current immediate need for an accepted *post-COVID-19 condition* COS, the development of a COS for the *post-COVID-19 condition* could act as a guide over the next few years until more is known and/or review or reassessment is indicated.

## Post-COVID condition health consequences

Recent editorials [4–6] and National Institutes of Health (NIH) [7] and WHO [8] sponsored conferences have drawn attention to an increasing number of people experiencing health consequences following the acute phase of SARS-CoV-2 infection and are calling for research into the risk factors, clinical features, diagnosis, management, and outcomes. Increasing funding opportunities have subsequently followed [9, 10]. It is important to note that most data regarding *post-COVID-19 condition* have been generated prior to the condition definition announcement. Thus, earlier studies may not fit the proposed definition criteria. *Post-COVID-19 condition* extends beyond the cardio-respiratory system to affect most other bodily systems both anatomically and physiologically [11]. Although causes of *post-COVID-19 condition* are unclear, persistent immune activation may be involved [12]. Risk factors for different syndromes of post-acute SARS-CoV-2 sequela have not been characterised, but it has been hypothesised that several *post-COVID-19 condition* phenotypes may exist, although pathophysiology, management, and outcomes are currently unknown.

Long-term health consequences of COVID-19 remain unknown, but reports suggest that prolonged symptom duration and limitations in functioning are common among hospitalised as well as non-hospitalised adults [13, 14] and children [15, 16]. The spectrum of long-lasting symptoms is wide and varies from mild discomfort to severe adverse effects on physical, cognitive, and psychosocial health [17], with important wider implications on functioning, including employment and school attendance.

Multiple studies from different countries found that many individuals experienced persistent symptoms 6 months after COVID-19, with fatigue or muscle weakness, sleep difficulties, and anxiety or depression among the most common sequelae [13, 14, 18]. A recent study suggests that although most COVID-19 survivors recover both physically and functionally a year after acute infection, some still experience problems with mobility, pain or discomfort, and anxiety or depression compared with non-COVID-19 controls [19]. The data emerging from the controlled studies are in agreement with the earlier reports. A recent analysis of the data from over 250,000 electronic health records demonstrated that more than one in three individuals had one or more features of *post-COVID-19 condition* recorded between 3 and 6 months after a diagnosis of COVID-19, which was significantly higher when compared with individuals with influenza [20–24]. Disease severity, female sex, and younger age were associated with a higher risk of *post-COVID-19 condition* development.

Yet, it is unknown whether persistent symptoms and associated abnormalities will fully resolve or whether some may leave life-long dysfunction. It is also worth noting that investigations into the *post-COVID-19 condition* can be difficult, with high loss to follow-up, frequent use of unvalidated measurement instruments, lack of inclusion of controls during the pandemic, and censoring of data (e.g. for death) not always fully considered in published studies. Differential diagnosis can be challenging with specific symptoms attributed to *post-COVID-19 condition* being a sign of an ongoing problem (e.g. dysautonomia in people reporting heart rate variability) [25].

Investigation of potential *post-COVID-19 condition* treatment options is still in its early days. Approaches mainly focus on rehabilitation and symptomatic management. Some experts suggest that antibodies and T cells able to recognise SARS-CoV-2 induced by vaccine “may help the immune system to stop the virus during its first few replications before it can establish hidden reservoirs in the body” [26]; however, the evidence regarding the effectiveness of SARS-CoV-2 vaccines in *post-COVID-19 condition* treatment is somehow conflicting [27, 28]. One of the major obstacles in the development of intervention strategies for *post-COVID-19 condition* is the lack of agreed outcomes to be assessed in clinical trials.

The pandemic and subsequent mitigation strategies have also had a substantial impact on the psychosocial well-being of the general population worldwide, with many people experiencing anxiety and depression, due to isolation, economic instability, job insecurity, sickness/death of infected family members, COVID-19-related stigma, lack of trust in government agencies, and constant media attention focused on the pandemic threats [29]. Disruption of care for those with pre-existing conditions has also had a large impact. For example, according to the WHO *Pulse survey on continuity of essential health services during the COVID-19 pandemic* [30], 45% of countries still reported disruptions to services for mental, neurological, and substance use disorders in the first quarter of 2021. On a similar scale, rehabilitation services disruptions continue to be reported by 53% (of 89 countries).

In addition, indirect impacts of COVID-19 on mental health [31], psychosocial, and neurological sequelae have been reported in adults following COVID-19 [32], and many patients are facing a variety of consequences including fatigue, shortness of breath, and cognitive dysfunction as well as reduced quality of life [18] with an impact on everyday functioning, even months following acute infection [19].

With millions of people affected by COVID-19, even a small percentage developing the *post-COVID-19 condition* will result in a detrimental effect on society and public health, with many people in need of long-term follow-up, management, and support[5]. A recent study has reported that previously hospitalised patients with COVID-19 had increased rates of multiorgan dysfunction compared with the general population [33].

## Terminology and clinical definitions

With many unresolved issues regarding this condition, the inclusion of patients’ perspectives has become increasingly important to the development of a COS. Importantly, there is currently no agreement on a clinical definition and which outcomes should be measured and how they should be measured. WHO has recently completed a Delphi consensus to finalise a clinical case definition of post-COVID-19 condition as described below [34]. This official WHO definition was published recently and is formulated as the following: “post-COVID-19 condition occurs in individuals with a history of probable or confirmed SARS CoV-2 infection, usually 3 months from the onset of COVID-19 with symptoms and that last for at least 2 months and cannot be explained by an alternative diagnosis. Common symptoms include fatigue, shortness of breath, and cognitive dysfunction, but also others and generally have an impact on everyday functioning. Symptoms may be new onset following the initial recovery from an acute COVID-19 episode or persist from the initial illness. Symptoms may also fluctuate or relapse over time” [35].

Other organisations have also proposed interim definitions such as the United Kingdom National Institute for Health and Care Excellence (NICE) who suggest an interim definition of post-COVID-19 syndrome as “signs and symptoms that develop during or after an infection consistent with COVID-19, continue for more than 12 weeks, and are not explained by alternative diagnoses” [36]. Due to the scarcity and high degree of heterogeneity of existing studies, the definition may change with the emergence of new evidence, but diversity in diagnostic criteria, methodology, and outcomes measured may slow down the progress. There is a need for terminology harmonisation, and consensus between major public health and government research organisations and professional bodies should be reached for the benefit and convenience of clinicians, researchers, and most importantly patients. With the growing evidence on COVID-19 consequences, there is a risk that the number of different terms used for the *post-COVID-19 condition* will increase further with increasing phenotyping of this condition.

## Core Outcome Set definition and relevance

Outcomes are measured in clinical research or practice to help evaluate relevant associations, safety and efficacy, risk factors, and effectiveness of interventions [37]. The lack of agreed upon outcomes and associated measurement instruments may complicate the evidence synthesis due to the inability to pool data in meta-analyses [38], resulting in a heterogeneous, incomplete, and low-quality evidence base and a barrier to clinical guideline development and policymaking. A COS comprises a minimum list of outcomes recommended for assessment in all studies, clinical practice setting, or both, for a specific condition and is necessary to harmonise research outputs and increase their comparability, quality, and generalisability to ensure their relevance to all interested partners. The failure to consider the views of patients and their families in selecting outcomes may result in less relevant outcome measures being evaluated, while the most important outcomes may be missed.

## Core Outcome Sets for COVID-19 and *post-COVID-19 condition*

A number of COVID-19-related COS are registered at the Core Outcome Measures in Effectiveness Trials (COMET) Initiative web-registry of planned, ongoing, and completed COS studies [39]. These initiatives provide clinicians, researchers, and policymakers with important information on the relevant aspects of COVID-19 and allow for the generation of harmonised high-quality data. Notably, COVID-19 COS development projects were undertaken in a rapid fashion, in contrast with the usual COS development process which typically takes several years.

Although none of the available COVID-19-related COS projects is solely dedicated to the *post-COVID-19 condition*, some include outcomes for “rehabilitation period”, “longer term impacts”, and “recovery” outcomes [40, 41]. Despite a well-established and standardised approach to COS development, which is generally guided by the COMET Handbook, the development of COS for the *post-COVID-19 condition* may be a complex task given the diversity and multisystem nature of infection sequelae [33]. COS previously developed for other conditions, which may be relevant for people recovering from COVID-19, should be considered a potential option for certain groups of individuals (e.g. acute respiratory failure/acute respiratory distress syndrome survivors after hospital discharge) [42]. We reviewed studies; assessed in the living systematic review of *long COVID* [43], data from the clinical trial registries, and available case report forms; and outlined outcomes previously measured in *long COVID* studies (Table 1).

**Table 1:** The long list of outcomes used in post-COVID-19 condition studies

Outcome area	Outcome domain (per COMET taxonomy)	Outcome
**Death**	1. Mortality/survival	All-cause mortality
**Physiological/clinical**	2. Blood and lymphatic system outcomes	Sustained prothrombotic changes
		Anaemia
		Thrombocytopenia
		Neutrophil to lymphocyte ratio changes
		Changes in inflammatory markers
		Changes in serum creatine kinase (CK)
		Changes in lactate dehydrogenase (LDH)
		Changes in glutamic-pyruvic transaminase (GPT)
		Electrolytes changes
	3. Cardiac outcomes	Angina pectoris
		Acute coronary disease
		Heart rhythm issues
		Heart failure
		Palpitations
		Chest tightness
		Newly diagnosed hypertension
		Myocardial fibrosis
		Myo- or pericarditis
		Changes in cardiovascular fitness
		Signal variations in the electrocardiogram (ECG)
		High blood pressure
	4. Endocrine outcomes	Diabetes mellitus
		Worsening control of existing diabetes (T1/T2)
		Diabetic ketoacidosis
		Hyperlipidaemia
		Subacute thyroiditis
		Hyperthyroidism
		Hypothalamic-pituitary-adrenal axis suppression
	5. Ear and labyrinth outcomes	Tinnitus
		Hearing problems
	6. Eye outcomes	Visual disturbance
		Red eyes/eye irritation
		Conjunctivitis
		Dry eye disease
		Sicca syndrome
	7. Gastrointestinal outcomes	Nausea or vomiting
		Diarrhoea
		Gastritis
		Dyspepsia
		Gastro-oesophageal reflux disease (GORD)
		Dysphagia
		Bloody stool
		Enrichment of opportunistic organisms and depletion of beneficial commensals
		Post-infectious irritable bowel syndrome
		Constipation
	8. General outcomes	Fatigue
		Fever
		Malaise
		Weakness
		New daytime sweating
		New night sweats
		Flushing
		Loss of appetite
		Hair loss
		Unspecified pain
		Sleep disorder
		Chest pain
		Breathlessness
		Sleep apnea
		Voice change
		Abdominal pain
		Faints
		Limb oedema
		Dry mouth
		Dental issues
	9. Hepatobiliary outcomes	Chronic liver disease
		Liver function test changes
	10. Immune system outcomes	Hyperinflammatory state-induced SARS-CoV-2
		Post-MIS-C: coronary artery aneurysm, neurologic (headache, encephalopathy, stroke and seizure) complications
	11. Infection and infestation outcomes	Prolonged viral faecal shedding
		Tuberculosis
	12. Metabolism and nutrition outcomes	Unintentional weight loss
		Unintentional weight gain
		New-onset bone demineralisation
		Unintentional change in body constitution
	13. Musculoskeletal and connective tissue outcomes	Myalgia
		Arthralgia
		Limb pain—upper or lower
		Muscle atrophy
		Changes in neuromuscular performance during resistance exercise
		Dorsal/low back pain
	14. Outcomes relating to neoplasms: benign, malignant, and unspecified (including cysts and polyps)	Worsening of pre-existing cancer/neoplasm
	15. Nervous system outcomes	Dizziness
		Headache
		Stroke
		Autonomic dysfunction
		Tremors
		Seizures
		Taste disturbance
		Smell disturbance
		Bradykinesia
		Dysmetria
		Speech difficulty/dysarthria
		Numbness
		Guillain-Barré syndrome
		Abnormal reflex status
		Trigeminal neuralgia
		Neuralgia/neuropathy
		Frontal release signs
		Parkinsonism
		Problems with balance
		Encephalitis
		Brain physiology changes
		Restless legs
		Abnormal muscle tone
	16. Renal and urinary outcomes	New-onset bladder incontinence
		Acute kidney injury
		Chronic kidney disease
		Urinary tract infections
		Problems passing urine
		Microhaematuria
		Renal function tests change
		COVID-19-associated nephropathy (COVAN)
	17. Reproductive system and breast outcomes	Dysmenorrhea
		Erectile dysfunction
		Semen/sperm changes
		Infertility
	18. Psychiatric outcomes	Depression
		Anxiety
		Post-traumatic stress disorder (PTSD)
		Acute stress discorder
		Mood change
		Obsessive-Compulsive Disorder (OCD)
		Behaviour change
		Thoughts of self-harm/suicide
		Risk to self and/or others
		Psychosis
		Traumatic bereavement
		Substance abuse
		Smoking habit
		Hallucinations
	19. Respiratory, thoracic, and mediastinal outcomes	Sore throat
		Sneezing
		New-onset Chronic obstructive pulmonary disease (COPD)
		Excessive sputum expectoration
		Nasal congestion
		Catarrh
		Wheezing
		Cough
		Lung fibrosis
		Pleurisy
		Pleural effusion
		Pain on breathing
		Pulmonary function abnormalities
		Hypoxaemia
		Respiratory failure
		Respiratory disease
		Bronchiectasis
		Asthma
	20. Skin and subcutaneous tissue outcomes	Ulcers
		Skin rash
	21. Vascular outcomes	Thromboembolism
		Venous thrombosis
		Pulmonary and systemic vascular disease
	22. Congenital, familial, and genetic outcomes	
	23. Pregnancy and puerperium and perinatal outcomes, including breastfeeding and weaning	
**Life impact**	24. Physical functioning	Post-exertional malaise
		Impaired mobility
		Walking/gait abnormality
		Problems with usual activities
	25. Social functioning	COVID-related relationship issues
	26. Role functioning	Functioning
		Work/occupational function changes
	27. Emotional functioning/well-being	Demoralisation symptoms
		Coping issues
		Low mood
		Burnout
		Perceived stigma/discrimination
		Worry about infecting others
		Worry about invasion of privacy
		Need for accurate information from the government
		Fear of no full recovery
	28. Cognitive functioning	Confusion
		Concentration impairment
		Memory impairment
	29. Global quality of life	Reduced quality of life
		Reduction in health-related quality of life scores
	30. Perceived health status	Illness perceptions
	31. Delivery of care	Lack of information/uncertain prognosis
		Difficulty accessing and navigating services
		Difficulty being taken seriously/achieving a diagnosis
		Variation in standards (e.g. inconsistent criteria for seeing, investigating, and referring patients)
		Variable quality of the therapeutic relationship
	32. Personal circumstances	Self-care ability
		COVID-related life issues such as debt, unemployment, and family relationships
		Personal finances difficulties
**Resource use**	33. Economic	Health economic
	34. Hospital	Post-intensive care syndrome
	35. Need for further intervention	Hospital readmission
		Further healthcare contact
		Lung transplantation
		Oxygen dependence
		RRT requirement
		Need for regular medical check-ups after discharge
		Need for psychiatric service
	36. Societal/carer burden	Care dependency
		Carer burden
**Adverse events**	37. Adverse events/effects	Vaccination adverse effects
		Adverse effects of prednisolone

## Considerations and limitations within vulnerable populations

The development of a COS in vulnerable populations such as people with disabilities/comorbidities, undergoing complex treatment (e.g. chemotherapy or transplantation), elderly, racial and ethnic minority groups, pregnant women, and children have special challenges that merit additional considerations. These challenges revolve not just around the constellation of symptoms but also the right time for the assessment and the individual on whom these are assessed. For a pregnant woman who was infected in the first trimester and recovered fully before delivery, any adverse birth outcome, such as preterm birth, stillbirth, or pregnancy complication (e.g. preeclampsia or gestational diabetes), may or may not be part of the *post-COVID-19 condition* [44]. Whether the congenital anomalies in the neonate or any neonatal complication following maternal COVID-19 can be defined as a potential *post-COVID-19 condition* for the neonate or infant requires consideration. It is particularly important to differentiate an adverse birth outcome that could be attributed to maternal COVID-19 from one that would have occurred otherwise due to other risk factors, irrespective of maternal COVID-19 status. This is similar to ruling out other aetiologies within the general population (non-pregnant adults).

## Considerations and limitations within low-middle income settings

There are multiple implications to the COVID-19 pandemic on low- and middle-income countries (LMICs) including lack of available healthcare resources to meet the needs of the local population, not only with COVID-19 infection, but for other acute and chronic conditions as well [45]. Another anticipated challenge for LMICs is the recognition of the *post-COVID-19 condition*. Post-acute care of physical, cognitive, and mental health disabilities may go under-recognised, especially in low-resource contexts in which all efforts are focused on containing COVID-19 dissemination and providing an appropriate care for severely ill patients. This may impact research on the *post-COVID-19 condition* as well as identification and management in LMICs.

Current definitions for the assessment of specific outcomes involve advanced laboratory and imaging techniques which require resources and skills. Such skills and resources may not be readily available in resource-limited settings. An inclusive approach should be taken while compiling the COS, by including alternate definitions and methods of measurement which may be acceptable for low-middle income settings. Extraordinary care must be taken to strike a balance between accuracy of the assessment and feasibility across the globe. The development of a COS for *post-COVID-19 condition* should account for cultural and social differences and restrictions in access and resources. Clinicians, researchers, and patient representatives from LMICs should be engaged in the COS development process to ensure global representability and future applicability of the COS.

## Limitations to existing research

Although attention to the problem of the *post-COVID-19 condition* is increasing, there are still many unanswered questions and important limitations impacting research quality and understanding of COVID-19 sequelae.

We outline these as well as potential mitigation strategies following a systematic approach [3] or defining research priorities through planning, implementation, publishing, and evaluation phases in Table 2. The table was drafted by DM and critically appraised and approved by all the authors. *Post-COVID-19 condition* still has no consensus definition, well-defined clinical phenotypes, or clearly explained underlying physiological mechanisms. WHO has highlighted “three Rs” related to *post-COVID-19 condition*—recognition, research, and rehabilitation, and initiated working groups aiming to provide a *post-COVID-19 condition* clinical definition [46] and outline plausible explanations of the physiological mechanisms as well as proposed an interim clinical case definition through a multi-disciplinary, gender-based, international Delphi consensus [34].

**Table 2:** Existing limitations of post-COVID-19 condition research and potential mitigation strategies

	Issue	Potential mitigation strategies
1.	The definition of *the post-COVID-19 condition.*	A few initiatives were launched, including a WHO working group aiming to provide a clinical case definition of *the post-COVID-19 condition*.
2.	Pathophysiological mechanisms still lacking.	A WHO working group has been set up to outline plausible hypotheses regarding the underlying immunological and physiological mechanisms of *post-COVID condition*. Multinational studies aimed to dissect the underling mechanisms of *the post-COVID-19 condition* should be lunched by multilateral organisations and universities.
3.	Rapidly emerging data.	Core Outcome Set (COS) should be developed keeping the balance between speed and quality. Acute COVID-19 COS initiatives [41] may be used as an example of efficient management and rapid development. Involve principle investigators from ongoing studies to investigate the possible additional sources of data and allow for the dissemination of the COS upon development. Close interaction and collaboration with the WHO to ensure global geographical coverage and worldwide applicability of the COS.
4.	Target population and scope: ● Hospitalised cohorts may be potentially different to those studies investigating non-hospitalised patients. ● Criteria for hospitalisation vary substantially within hospitals and countries (i.e. hospitalised patients are different). ● The need for a separate *Post-COVID-19 condition* COS development for children has not been established.	Despite the focus of COVID-19 research on adults, all age and severity (during acute phase) groups (including asymptomatic individuals) should be included (approaches to patient routing differ within and between the countries and criteria for hospital admission vary). It is imperative to develop COS for children and their carers as *the post-COVID-19 condition* may potentially have a detrimental life-long effect on child health. A single COS aiming at clinical as well as research settings may be developed.
5.	What to measure*?* ● There is a need to define which data should be assessed in the trials and in clinical practice.	Ongoing systematic reviews may assist with the development of a list of candidate outcomes for the evaluation as part of a Core Outcome Set. Patient engagement should drive the agenda to ensure that patient-important outcomes are captured. This can be achieved by survey/Delphi process and consensus meetings. *Post-COVID-19 condition* COS development is a priority.
6.	How to measure *the Post-COVID-19 condition?* ● Existing “validated” tools (e.g. quality of life instruments) have not been validated in COVID-19, and study participants are often asked about their experience pre-COVID retrospectively, which may lead to selection and recall bias. ● New measurement instruments may need to be developed if no adequate instruments exist for prioritised Core Outcome Domains.	Measurement instruments used in the studies should be systematically reviewed and assessed for validity/truth, discrimination ability, and feasibility.

The number of studies assessing *post-COVID-19 condition* is increasing, generating a large amount of data, where validity remains unknown. There is a large variability in reporting and quantification of post-COVID-19 condition symptoms among the studies. It is important to note that not only symptom presence is essential, but symptom duration and severity also merit consideration. The lack of pre-morbid data for comparison is one of the major limitations of *post-COVID-19 condition* research. Any abnormalities found are normally attributed to *post-COVID-19 condition* assuming that the patient did not have asymptomatic abnormal testing before infection.

Few international initiatives have created instruments for data collection. The International Severe Acute Respiratory and Emerging Infection Consortium (ISARIC) has developed follow-up protocols and surveys for adults and children to assess the prevalence and risk factors for long-term physical and psychosocial health consequences following COVID-19 diagnosis. A *post-COVID-19 condition* case report form has been designed by the WHO to report standardised clinical data from individuals after hospital discharge or after the acute illness to examine the medium- and long-term consequences of COVID-19. Although these instruments assist harmonised data collection, an increasing number of tools from reputable organisations may result in data heterogeneity with different centres prioritising different instruments.

There are few ongoing initiatives tackling the problem of data heterogeneity by systematically reviewing available evidence in the live format [43], which may inform COS initiatives and assist with the long list of outcome development. However, systematic reviews will not address the problem of instrument validity. Assessment of the validity may take a long time, and meanwhile, a COS should be developed.

A significant gap and limitation within *post-COVID-19 condition* research exist within paediatric and adolescent development considering that life-long consequences may exist [47]. Outcomes of interest in children and adolescents may be very different to the adult population, and COS for this age group should be specifically developed engaging the children and adolescents themselves, as well as their parents and carers.

## Conclusions

This manuscript was written by a multidisciplinary (allergists, critical care specialists, ENT specialists, infectionists, immunologists, neurologists, psychiatrists, paediatricians, pulmonologists, specialists in global and public health experts, epidemiologists, methodologists, rehabilitation specialists, and people with lived experience of *post-COVID-19 condition*), gender-balanced, international group of experts, including members of the ISARIC Consortium, US Centers for Disease Control and Prevention (CDC), experts involved in the WHO *post-COVID condition* clinical characterisation group, leads of international COVID-19 cohorts, members of Core Outcome Measures for *post-COVID-19 condition/long COVID* initiative and patient representatives, to outline the unmet needs and justification for Core Outcome Set development for the *post-COVID-19 condition* which may become a major public health burden. Previous research in various medical fields has demonstrated the importance and usefulness of COS in both research and clinical practice. There is a need to rapidly develop a COS for the *post-COVID-19 condition* which will allow for the improvement in data quality, harmonisation, and comparability between different geographical locations. The joint initiative requires input from all relevant partners, including, but not limited to, healthcare professionals, researchers, methodologists, patients, and carers. We urge local and international funding agencies to provide support for coordinated actions aiming to develop COS for *post-COVID-19 condition* in adults and children.

## Data Availability

Not applicable.

## Abbreviations

CDC: US Centers for Disease Control and Prevention
COMET: Core Outcome Measures in Effectiveness Trials Initiative
COS: Core Outcome Set
COVID-19: Coronavirus disease 2019
ISARIC: International Severe Acute Respiratory and Emerging Infection Consortium
LMICs: Low- and middle-income countries
NICE: National Institute for Health and Care Excellence
NIH: National Institutes of Health
PASC: Post-acute sequelae of SARS-CoV-2 infection
SARS-CoV-2: Severe acute respiratory syndrome-related coronavirus 2
WHO: World Health Organization
